# Causal relationship between hypothyroidism and coronary atherosclerotic cardiovascular disease: a bidirectional two-sample Mendelian randomization

**DOI:** 10.3389/fcvm.2024.1402359

**Published:** 2024-10-24

**Authors:** Jiarui Li, Yihan Wang, Xiaoting Luo, Tianwei Meng, Chengjia Li, Juan Li, Likun Du

**Affiliations:** ^1^ Heilongjiang University of Chinese Medicine, Harbin, China; ^2^ First Affiliated Hospital, Heilongjiang University of Chinese Medicine, Harbin, China; ^3^ The First Affiliated Hospital of Harbin Medical University, Harbin, China

**Keywords:** hypothyroidism, atherosclerotic cardiovascular disease, Mendelian randomization, enrichment analysis, causal relationship

## Abstract

**Background:**

Epidemiological and observational studies demonstrate a comorbid relationship between hypothyroidism and atherosclerotic cardiovascular disease (ASCVD). The nature and direction of this causal relationship, however, remain unclear.

**Objective:**

This study aims to elucidate the causal relationship between hypothyroidism and ASCVD using a bidirectional Mendelian randomization approach.

**Method:**

Single nucleotide polymorphisms (SNPs) associated with hypothyroidism were identified and selected as genetic instrumental variables from aggregated data of genome-wide association studies (GWAS). The outcome of interest, ASCVD, included seven conditions: coronary artery disease (CAD), angina pectoris (AP), myocardial infarction (MI), ischemic stroke (IS), and subtypes IS-large artery atherosclerosis (IS-LAA), IS-small vessels (IS-SV), and peripheral artery disease (PAD). MR analysis employed multiple methods—chiefly inverse variance weighting (IVW), along with MR Egger, weighted median, and weighted mode—to assess causality. Cochrane's *Q* test was utilized to evaluate heterogeneity in the MR findings. Causal association reliability was assessed using the MR-Egger intercept, MR-PRESSO tests, and leave-one-out analysis. Reverse MR analysis ensued if forward MR identified a positive exposure-outcome association. Moreover, the DAVID database facilitated GO functional and KEGG pathway enrichment analyses of neighboring genes to instrumental variables, exploring potential disease mechanisms.

**Result:**

GWAS pooled data yielded 122 SNPs as potential instrumental variables for hypothyroidism. Forward MR analysis, using the IVW method, indicated hypothyroidism as a risk factor for CAD (OR = 2.34, 95% CI = 1.39–3.94, *P* = 0.001), AP (OR = 2.01, 95% CI = 1.28–3.16, *P* = 0.002), MI (OR = 1.02, 95% CI = 1.01–1.04, *P* = 0.004), and IS-SV (OR = 6.92, 95% CI = 2.45–19.55, *P* < 0.001). However, no significant link was found between hypothyroidism and the remaining three diseases, with sensitivity analysis reinforcing result robustness. In contrast, reverse MR analysis did not corroborate a causal link from ASCVD to hypothyroidism. The R package identified 83 neighboring genes as instrumental variables. GO enrichment analysis via the DAVID database yielded 53 entries, predominantly involving cAMP catabolic processes, protein binding, and signal transduction. KEGG analysis identified 31 pathways, notably those related to Th1/Th2 and Th17 cell differentiation, and Herpes simplex virus 1 infection.

**Conclusion:**

The marked association between hypothyroidism and CAD, AP, MI, and IS suggests that thyroid function assessment could be integral to preventing and diagnosing specific ASCVD types. This underscores the need for individuals with hypothyroidism to be proactive regarding ASCVD risk factors. A balanced Th1/Th2 and Th17/Treg ratio may offer a novel strategy in preventing CAD and enhancing the prognosis for hypothyroid patients.

## 1 Introduction

### 1.1 Atherosclerotic cardiovascular disease

ASCVD comprises conditions such as ischemic heart disease, ischemic stroke (IS), and peripheral arterial disease, all of which arise from arterial plaque accumulation. Notably, cardiovascular diseases (CVDs) are increasingly prevalent (1), resulting in approximately 17.9 million deaths each year, or 32% (2) of global mortality. Coronary artery disease (CAD), a form of ASCVD, is particularly significant; it develops from the narrowing of coronary arteries and is a leading cause of death worldwide. IS, primarily presenting as cerebral infarctions, are classified by their etiology: large artery atherosclerosis, small vessel disease, or cardiogenic embolism (3). Such strokes impose a considerable toll on health, especially in terms of morbidity and mortality among middle-aged and elderly populations (4).

### 1.2 Hypothyroidism

Hypothyroidism is characterized by high concentrations of thyroid-stimulating hormone (TSH) with concomitant low concentrations of thyroid hormones (Clinical Overt primary hypothyroidism) or within the reference range (Subclinical hypothyroidism). As the most common autoimmune disorder in adults, hypothyroidism is marked by mucopolysaccharide accumulation within tissues and the skin, with symptoms including fatigue, constipation, and cold intolerance. Thyroid function generally decreases with age; the prevalence of hypothyroidism is about 5.1% in women between 45 and 64 years, increasing to 12.7% in those over 65 (5).

### 1.3 Relevance studies

Thyroid hormones significantly influence cardiovascular health, with both clinical and subclinical hypothyroidism heightening CVD risk (6). These hormones affect cardiovascular function by modifying cardiac ion channels (7), altering macrophage inflammatory responses (8), and impacting mitochondrial function (9). Profound hypothyroidism can decrease stroke volume, heart rate, cardiac output, and myocardial contractility. Moreover, hypothyroidism impairs cardiovascular health by affecting left ventricular function and vascular compliance (10). Diminished thyroid hormone levels can lead to elevated homocysteine levels (an independent risk factor for ASCVD), dyslipidemia, especially elevated LDL-C (a major risk factor for atherosclerosis), systolic hypertension (a major cause of stroke), and hypercoagulable state of the blood, which can lead to pathological changes such as vascular endothelial damage and lipid deposition (11). Despite optimal management of known risk factors, residual CVD risk persists (12).

### 1.4 Mendelian randomization

Randomized controlled trials (RCTs) are the standard for establishing causality, and epidemiological studies seek to determine if risk factors can modify disease severity. However, traditional observational studies often fail to exclude confounding factors. Proposed by Katan in 1986, Mendelian randomization (MR) uses single-nucleotide polymorphisms (SNPs) as instrumental variables to ascertain causality. According to Mendel's laws of inheritance, capitalizing on the natural random assortment of alleles, a process similar to the randomized grouping process in RCTs, known as RCTs “in the state of nature”. This genetic approach minimizes confounding and reverse causation (13), and with the advent of large GWAS datasets, MR has flourished as a method to examine causal links in observational data.

Thus, this study employs MR to explore the causal relationship between hypothyroidism and ASCVD. Our findings provide a robust bioinformatics foundation for understanding the pathophysiology of combined hypothyroidism and ASCVD, and offer new perspectives for clinical prevention and treatment.

## 2 Data and methods

### 2.1 Sources of information

The source data for the exposure factor “hypothyroidism” were derived from a GWAS (ID: ukb-b-19732) by Ben Elsworth and colleagues in 2018. This study utilized the UK Biobank (UKB) cohort, comprising 22,687 patients and 440,246 controls, yielding 9,851,867 SNPs associated with hypothyroidism. GWAS data for the outcome factor “ASCVD” were obtained from the IEU OpenGWAS project (https://gwas.mrcieu.ac.uk/). The GWAS for CAD analyzed 29,339 cases and 322,724 controls, identifying 11,027,870 SNPs. The GWAS for AP involved 30,025 cases and 440,906 controls, yielding 24,170,313 SNPs. The MI GWAS comprised 11,081 cases and 473,517 controls, identifying 9,587,836 SNPs. The IS GWAS analyzed 11,929 cases and 472,192 controls, identifying 24,174,314 SNPs. The IS-LAA GWAS included 4,373 cases and 406,111 controls, yielding 7,992,739 SNPs. The IS-SV GWAS included 5,386 cases and 192,662 controls, identifying 6,150,261 SNPs. The PAD GWAS included 7,114 cases and 475,964 controls, identifying 24,186,090 SNPs. The selection criteria for each GWAS were based on the largest sample size and the most recent studies (Table 1). Ethical approvals and informed consent were secured for the original study, eliminating the need for additional ethical clearances.

**Table 1:** Details of the GWAS for outcome.

GWAS ID	Trait	N case	N control	Sample size	N snps	Population	Author	pubmed ID
ebi-a-GCST90013864	CAD	29,339	322,724	352063	11027870	European	Mbatchou J	34017140
ebi-a-GCST90018793	AP	30,025	440,906	470931	24170313	European	Ben Elsworth	34594039
ebi-a-GCST90038610	MI	11,081	473,517	484598	9587836	British	Handan Melike Dönertaş	33959723
ebi-a-GCST90018864	IS	11,929	472,192	484121	24174314	European	Sakaue S	34594039
ebi-a-GCST005840	IS-LAA	4,373	406,111	150765	7992739	European	Malik R	29531354
ebi-a-GCST005841	IS-SV	5,386	192,662	198048	6150261	European	Malik R	29531354
ebi-a-GCST90018890	PAD	7,114	475,964	483078	24186090	European	Sakaue S	34594039

### 2.2 Research methodology

#### 2.2.1 Selection of instrumental variables

**Figure 1:**
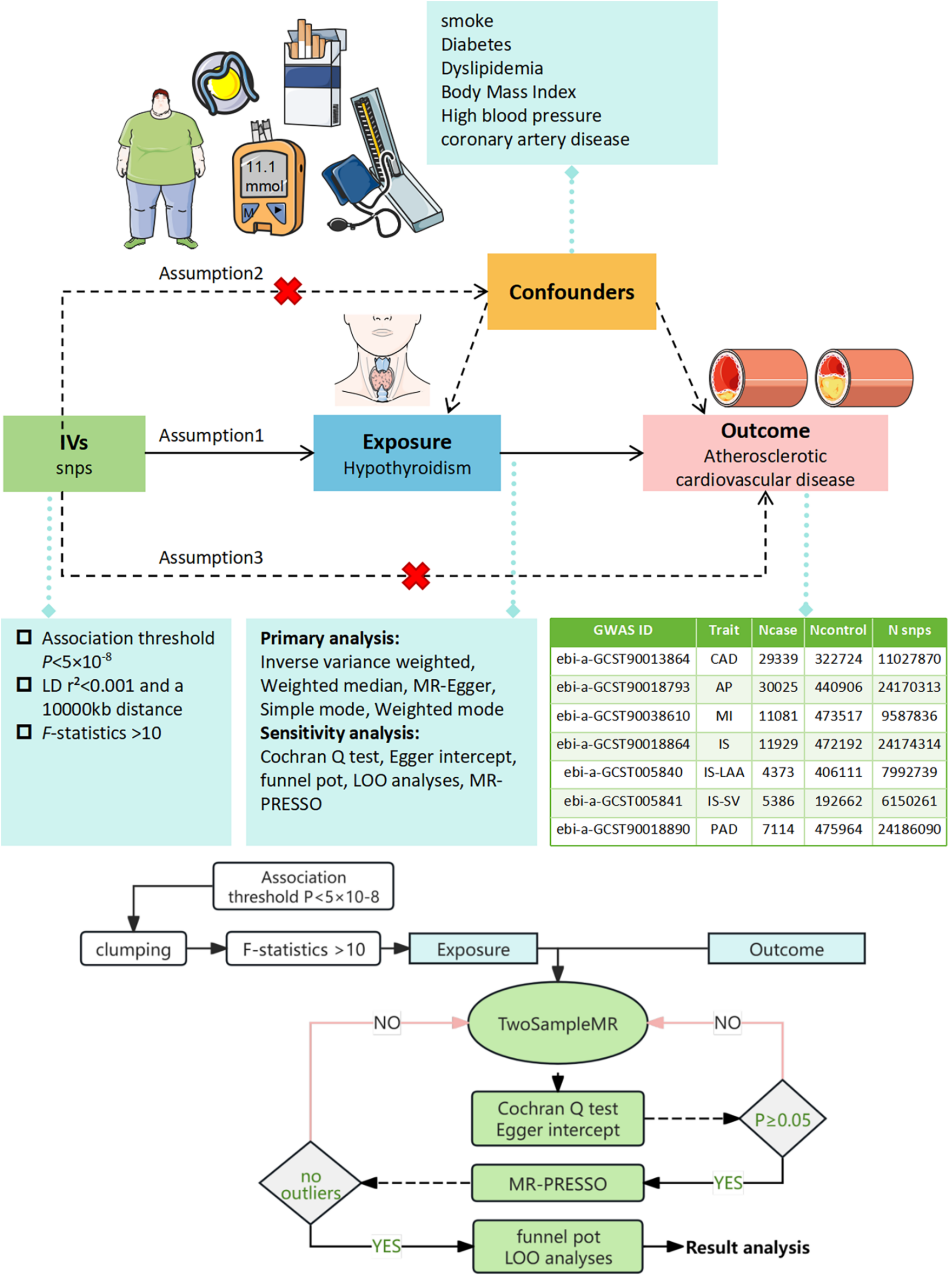
Illustration of the MR Framework for assessing the causal link between hypothyroidism and ASCVD. Assumption 1 posits that genetic variants are strongly associated with the exposure (hypothyroidism). Assumption 2 asserts that these genetic variants are not linked with any confounders. Assumption 3 states that the genetic variants influence the outcome exclusively through the exposure. SNPs, single nucleotide polymorphisms; LD, linkage disequilibrium; LOO, leave-one-out analysis; MR-PRESSO, MR Pleiotropy RESidual Sum and Outlier test.

To adhere to the MR criteria for instrumental variables, we: (I) selected SNPs significantly associated with hypothyroidism at *P* < 5 × 10^−8^ from the GWAS data, (II) identified independent SNPs using a linkage disequilibrium threshold of *r^2^* < 0.001 and a 1,000 kb window (Clumping), (III) ensured SNP effect alleles were consistent in both exposure and outcome GWAS (Harmonization), excluding those with ambiguous effect alleles and palindromic SNPs with intermediate allele frequencies, (IV) removed any SNPs linked to confounders for ASCVD utilizing Phenoscanner V2 (http://www.phenoscanner.medschl.cam.ac.uk/) with thresholds set at *P* < 5 × 10^−8^ and *r^2^* > 0.8, (V) excluded SNPs directly associated with ASCVD at *P* < 5 × 10^−8^, and (VI) computed the *F*-statistic for each instrumental variable, accepting those with *F* > 10 to avoid weak instrument bias (14), *F* = beta^2^/se^2^. The MR analysis process was conceptualized and illustrated as a directed acyclic graph (Figure 1).

#### 2.2.2 Two-sample MR analysis

We conducted a two-sample MR analysis to investigate the causal link between hypothyroidism and ASCVD using the “TwoSampleMR” package in R. The analysis applied various methods including MR-Egger, Weighted Median (WM), Inverse Variance Weighted (IVW), Simple Mode, and Weighted Mode, with IVW being the primary method. The IVW method yields precise causal effect estimates when all SNPs are valid instrumental variables (15). The WM method is reliable when over half of the SNPs are valid (16), while MR-Egger is used when there is suspicion that no SNPs are valid (17).

#### 2.2.3 Sensitivity analysis

We assessed heterogeneity using IVW and MR-Egger regression, quantifying it with Cochran's *Q* statistic and considering heterogeneity significant at *P* < 0.05, prompting the use of a random effects model (18). Horizontal pleiotropy was evaluated via the MR-Egger regression intercept; a significant non-zero intercept (*P* < 0.05) indicated its presence, rendering the MR results questionable at that point (16). MR-PRESSO was employed to detect and exclude outlier SNPs, after which the MR analysis was repeated to confirm result robustness (19). A LOO approach was implemented to determine the influence of individual SNPs on the exposure-outcome relationship.

#### 2.2.4 Reverse MR analysis

We conducted a bidirectional MR analysis to explore potential reverse causation. With ASCVD as the exposure and hypothyroidism as the outcome, we re-evaluated the direction of the causal relationship using the same data sources. Subgroup forest plots were used to present the results of these analyses.

#### 2.2.5 Enrichment analysis

To decipher the biological underpinnings of the implicated SNPs, we first identified proximal genes using the “vautils” package. When an SNP corresponded to multiple genes, the closest gene was selected. The list was then analyzed using the DAVID database (https://david.ncifcrf.gov/) for functional annotation, selecting “Homo sapiens” as the species and “official gene symbol” for gene names. Default parameters were applied to retrieve Biological Processes (BP), Molecular Functions (MF), Cellular Components (CC), and Kyoto Encyclopedia of Genes and Genomes (KEGG) pathway enrichment. Finally, the analysis results were visualized as GO function enrichment analysis matrix point plots (20) and KEGG enrichment analysis secondary classification summary plots (20) using an online graphing platform.

## 3 Result

### 3.1 Selection of instrumental variables

**Figure 2:**
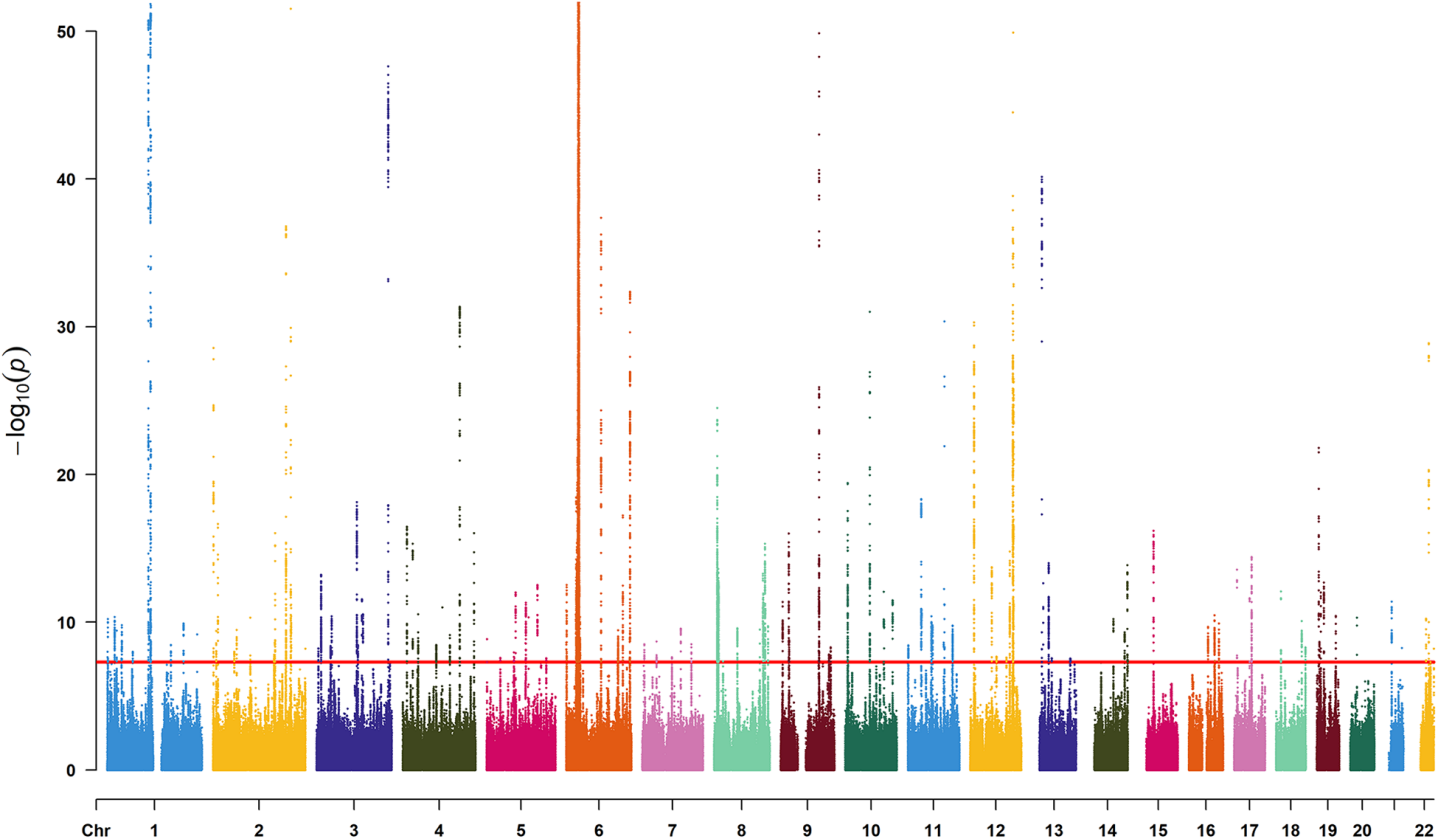
Manhattan plot for SNPs from the GWAS dataset “ukb-b-19732”. The *x*-axis denotes the chromosome numbers, while the *y*-axis represents the negative logarithm of each SNP's *P*-value, illustrating the strength of association with hypothyroidism—the lower the *P*-value, the higher the point on the plot. The red line indicates the significance threshold (*P* < 5 × 10^−8^).

**Figure 3:**
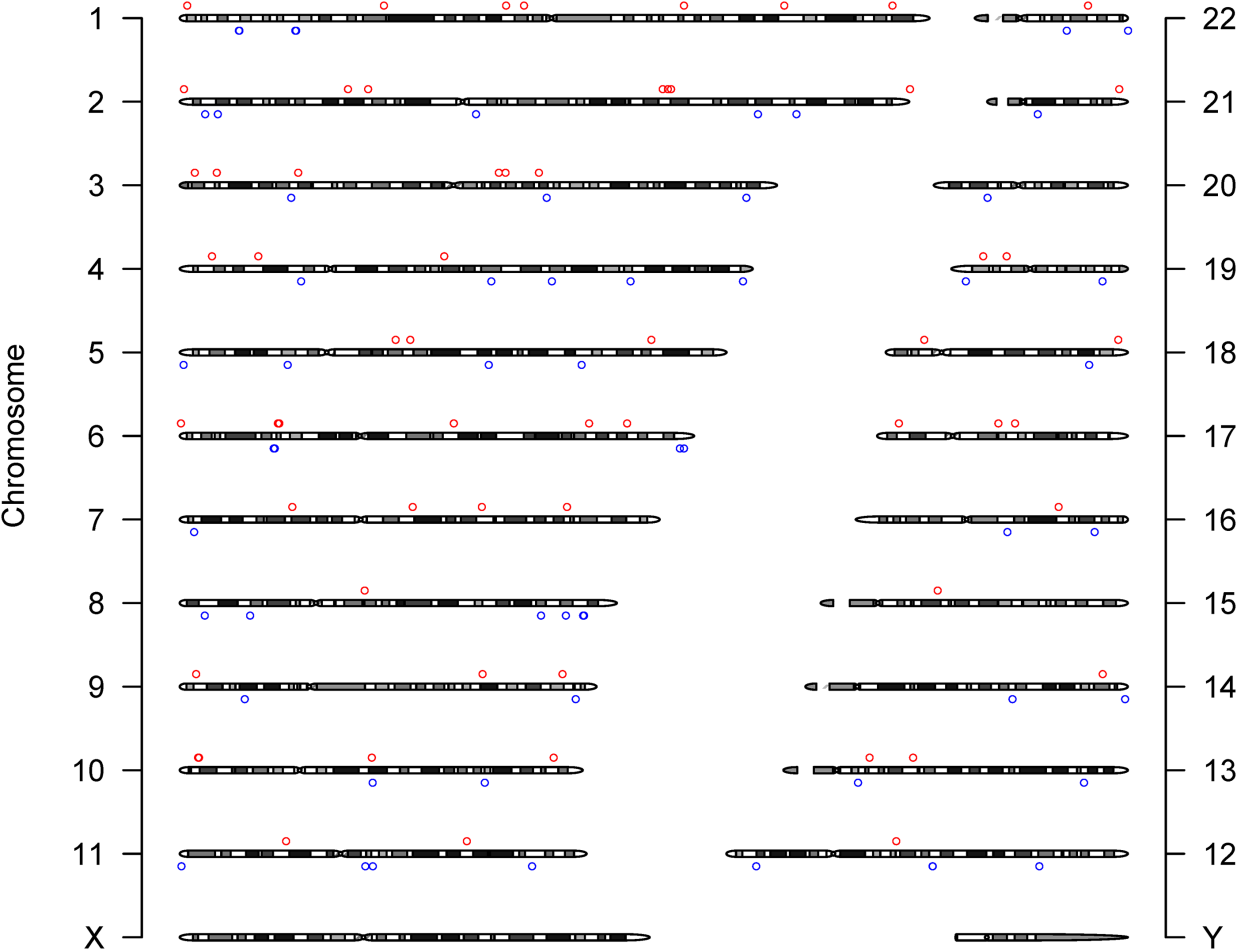
Chromosome distribution of 122 SNPs supporting the primary hypothesis. The *y*-axis labels the chromosomes. Circles (^○^) mark the chromosomal positions of SNPs: red^○^ for SNPs with a beta coefficient (effect value) greater than zero, blue^○^ for those with a beta less than zero, indicating their respective directional associations with hypothyroidism.

We identified 23,598 SNPs associated with hypothyroidism at a genome-wide significance level (*P* < 5 × 10^−8^) from a combined GWAS dataset. These associations were displayed in a Manhattan plot (Figure 2). Through linkage disequilibrium clumping, we extracted 122 independent SNPs associated with hypothyroidism, whose chromosomal distribution was visualized using an online mapping tool (20) (Figure 3). The top 20 SNPs with the lowest *P*-values are listed in Table 2. These SNPs were evaluated for association with seven ASCVDs, and no additional proxy SNPs were sought for any missing data during harmonization. SNPs correlating with confounders such as smoking, body mass index (21), hypertension, dyslipidemia, diabetes mellitus (22), CAD, and age were excluded (e.g., rs3184504, rs174599, rs7090530, rs3087243, rs28418426, rs2921053, rs772920, rs3850765, rs221781, rs2111485, rs731151). After outlier removal via the MR-PRESSO test, instrumental variables were confirmed. *F*-statistics for all instrumental variables exceeded 29, indicating no weak instrument bias (Table 3).

**Table 2:** Basic information on the top 20 differentially significant SNPs strongly associated with hypothyroidism.

SNP	Chr	Pos	EA	OA	EAF	Beta	SE	*P* Value	*F* statistics
rs9272426	6	32605189	G	A	0.4522	0.0125	0.0005	2.60 × 10^−168^	764.6930
rs6679677	1	114303808	A	C	0.1008	0.0202	0.0007	5.11 × 10^−167^	758.7276
rs3184504	12	111884608	C	T	0.5173	−0.0101	0.0004	3.00 × 10^−116^	525.3031
rs7850258	9	100549013	G	A	0.6680	0.0099	0.0005	1.90 × 10^−98^	443.4618
rs3087243	2	204738919	A	G	0.4507	−0.0086	0.0004	1.70 × 10^−82^	370.1398
rs28418426	6	32619654	C	T	0.5288	0.0091	0.0005	5.80 × 10^−79^	353.9570
rs7768019	6	31202575	G	C	0.2439	−0.0079	0.0005	1.20 × 10^−53^	237.7647
rs12634152	3	188121019	T	C	0.5473	−0.0065	0.0004	2.40 × 10^−48^	213.5122
rs9511151	13	24786576	A	G	0.3458	−0.0062	0.0005	1.10 × 10^−40^	178.4360
rs7754251	6	90989125	C	G	0.5798	0.0058	0.0004	4.20 × 10^−38^	166.5608
rs7582694	2	191970120	G	C	0.7739	−0.0068	0.0005	1.60 × 10^−37^	163.9122
rs9347170	6	167405187	T	C	0.3365	−0.0056	0.0005	4.30 × 10^−33^	143.6033
rs4835536	4	149662857	T	G	0.2126	−0.0064	0.0005	4.30 × 10^−32^	139.0354
rs9277569	6	33058402	T	C	0.1095	0.0083	0.0007	7.60 × 10^−32^	137.9278
rs71508903	10	63779871	T	C	0.1941	0.0066	0.0006	1.00 × 10^−31^	137.3601
rs4409785	11	95311422	C	T	0.1726	0.0068	0.0006	4.30 × 10^−31^	134.4721
rs11052877	12	9905690	G	A	0.3718	−0.0053	0.0005	5.00 × 10^−31^	134.1791
rs229540	22	37591290	G	T	0.4255	0.0051	0.0004	1.30 × 10^−29^	127.7641
rs11675342	2	1407628	T	C	0.4235	0.0050	0.0004	2.70 × 10^−29^	126.2377
rs2921053	8	8319963	C	G	0.4511	−0.0046	0.0004	3.10 × 10^−25^	107.6963

**Table 3:** Sensitivity analysis.

Outcome	Heterogeneity				Pleiotropy			Outlier test	
	MR Egger		IVW		MR egger intercept			MR Presso	
	Q-value	*P*-value	Q-value	*P*-value	Intercept	SE	*P*-value	Global test	*P*-value
CAD	108.706	0.099	110.201	0.095	−0.003	0.003	0.266	125.054	0.130
AP	127.709	0.065	127.720	0.074	−2.23 × 10^−4^	0.003	0.203	131.818	0.110
MI	114.361	0.250	116.635	0.226	−1.13 × 10^−4^	7.85 × 10^−5^	0.151	120.418	0.243
IS	131.087	0.057	131.303	0.063	−0.001	0.003	0.675	134.344	0.076
IS(LAA)	113.550	0.290	116.076	0.258	0.009	0.006	0.128	120.629	0.239
IS(SV)	96.453	0.328	97.880	0.318	0.007	0.006	0.249	101.931	0.331
PAD	126.210	0.078	128.616	0.067	0.006	0.004	0.160	131.441	0.080

SNP: single nucleotide polymorphism, CHR: chromosome number, POS: base location, EA: effector allele, OA: non-effector allele, EAF: effector allele frequency, Beta: allele effect size, SE: standard error of β, F-value: weak instrumental variable F-value

### 3.2 MR analysis results

**Figure 4:**
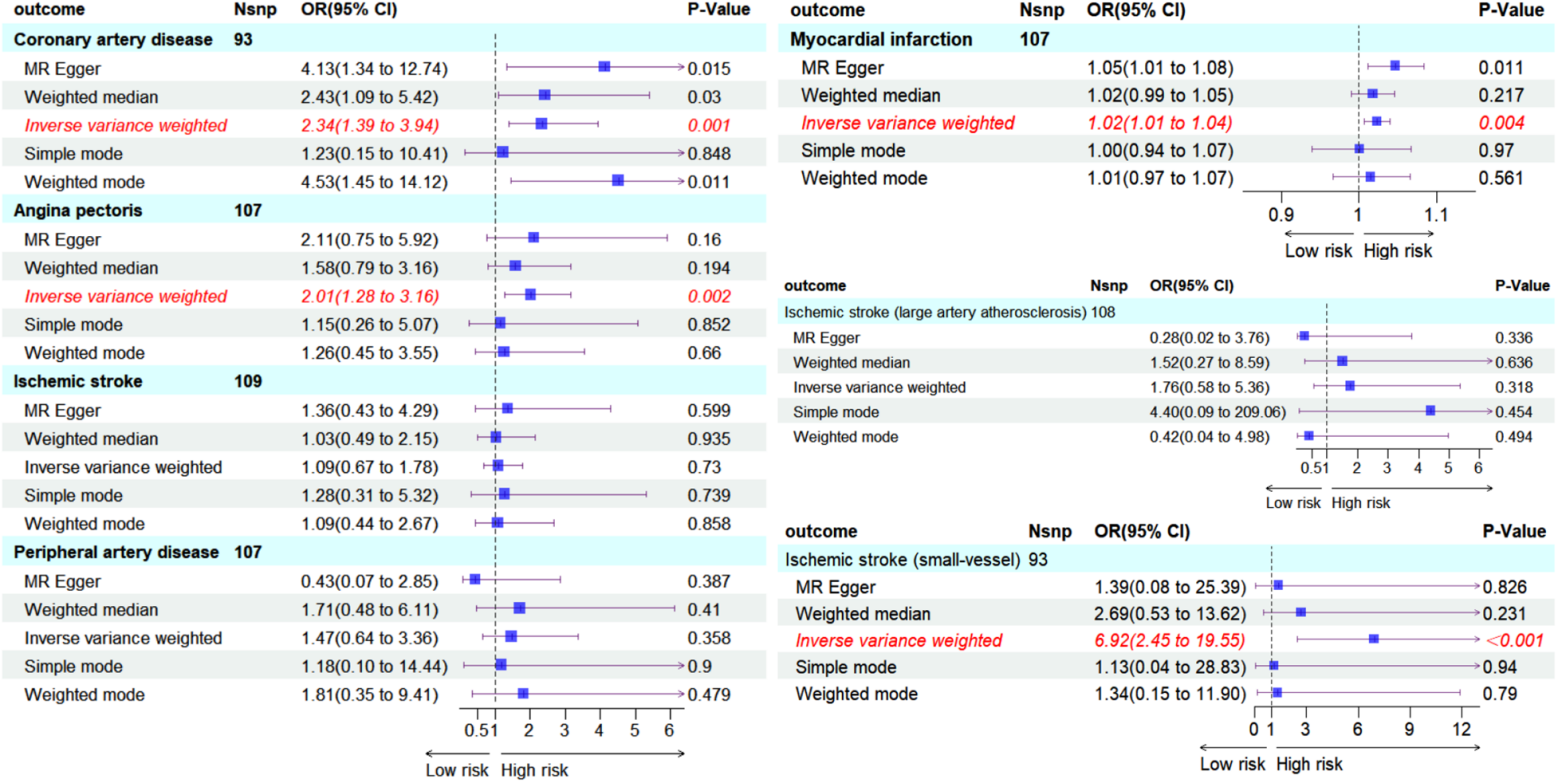
Subgroup forest plot depicting seven ASCVD outcomes and the count of SNPs used in their respective MR analyses, shaded in sky blue. Red italics highlight outcomes where the IVW method suggests a causal effect from hypothyroidism (*P* < 0.05). The IVW-based forward MR analysis indicates an increased risk of CAD, AP, MI, IS-SV due to hypothyroidism. No causal effect on IS, IS-LAA, PAD was observed.

We visualized the MR analysis results for the relationship between hypothyroidism and ASCVD in subgroup forest plots (Figure 4). Using the IVW method, the forward MR analysis indicated that hypothyroidism is associated with an increased risk of CAD (OR = 2.34, 95%CI = 1.39–3.94, *P* = 0.001), AP (OR = 2.01, 95%CI = 1.28–3.16, *P* = 0.002), MI (OR = 1.02, 95%CI = 1.01–1.04, *P* = 0.004), and IS-SV (OR = 6.92, 95%CI = 2.45–19.55, *P* < 0.001). Conversely, no association was found between hypothyroidism and overall IS (OR = 1.09, 95%CI = 0.67–1.78, *P* = 0.730), IS-LAA (OR = 1.76, 95%CI = 0.58–5.36, *P* = 0.318), or peripheral artery disease (PAD) (OR = 1.47, 95%CI = 0.64–3.36, *P* = 0.358).

### 3.3 Sensitivity analysis

**Figure 5:**
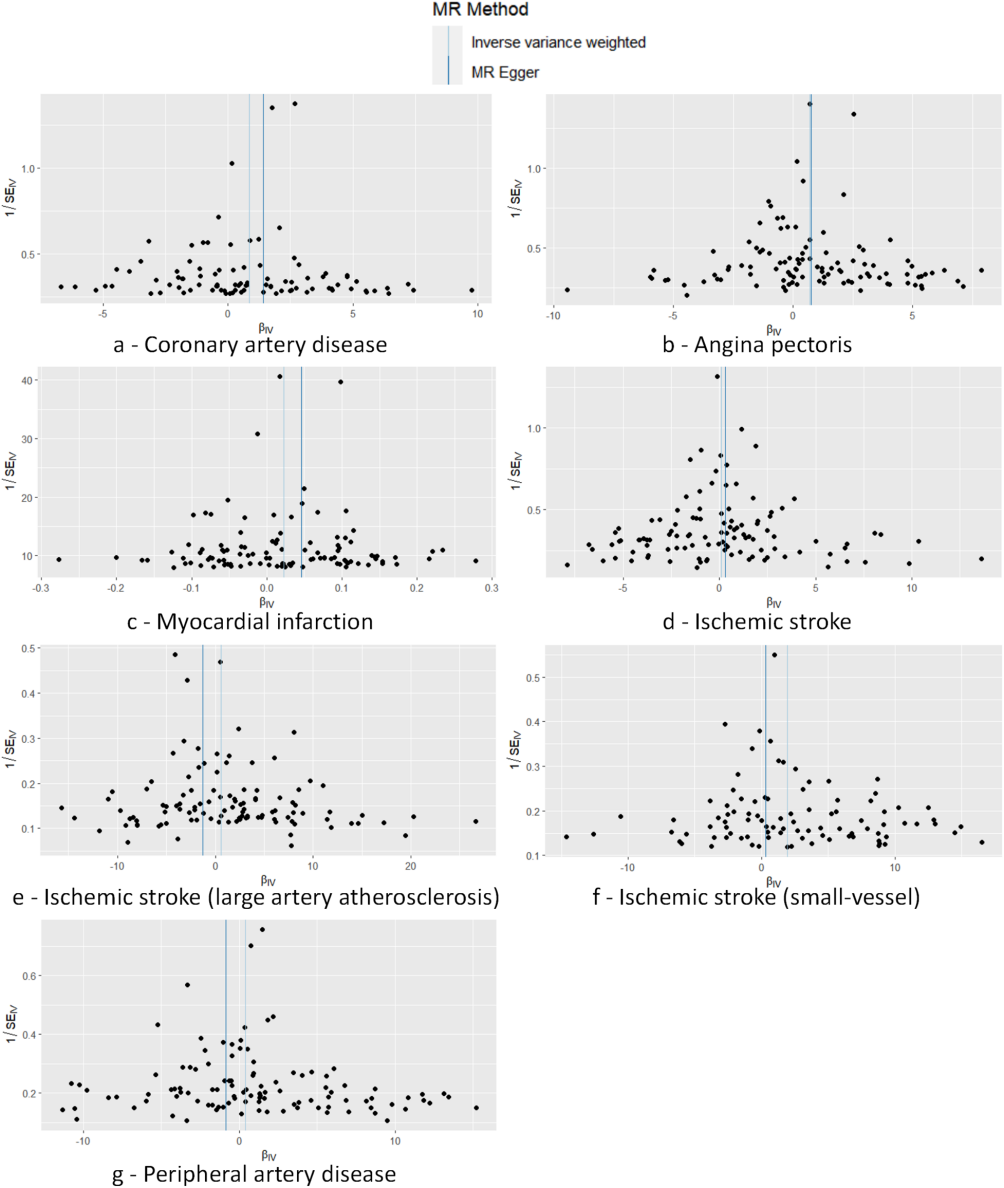
Funnel plot demonstrating symmetry across seven analyses, with no SNPs markedly deviating from the collective distribution. The exposure factors for part labels **(a–g)** are all hypothyroidism, with the outcome factors being: coronary artery disease (CAD), angina pectoris (AP), myocardial infarction (MI), ischemic stroke (IS), and subtypes including IS-large artery atherosclerosis (IS-LAA), IS-small vessels (IS-SV), and peripheral artery disease (PAD).

**Figure 6:**
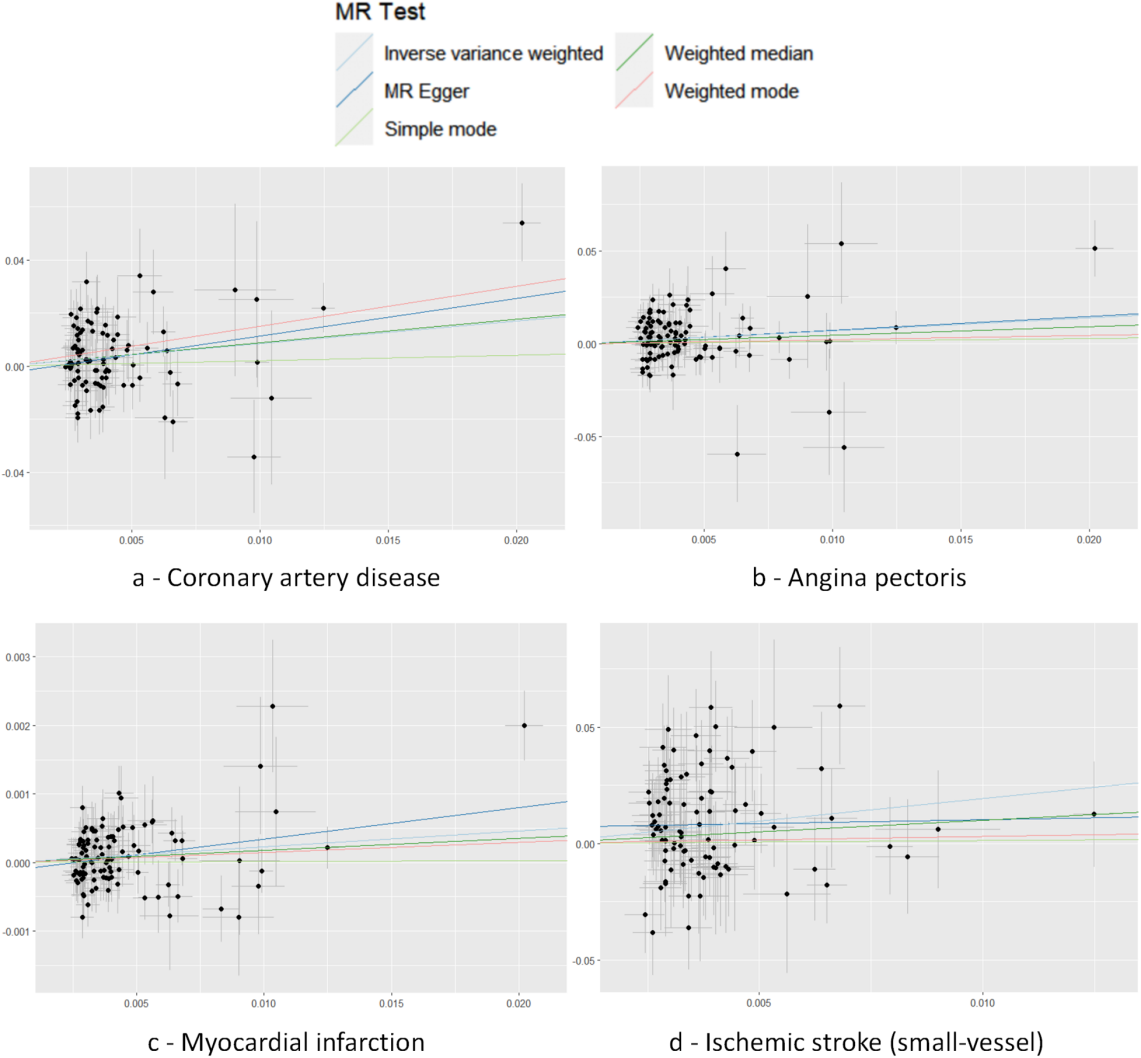
Scatter plot with each point representing an SNP. The accompanying line shows the 95% confidence interval. The *x*-axis measures the SNP's effect on the exposure, while the *y*-axis quantifies its effect on the outcome. Colored lines reflect the MR fitting results. The exposure factors for part labels **(a–d)** are all hypothyroidism, with the outcome factors being: coronary artery disease (CAD), angina pectoris (AP), myocardial infarction (MI), and IS-small vessels (IS-SV).

**Figure 7:**
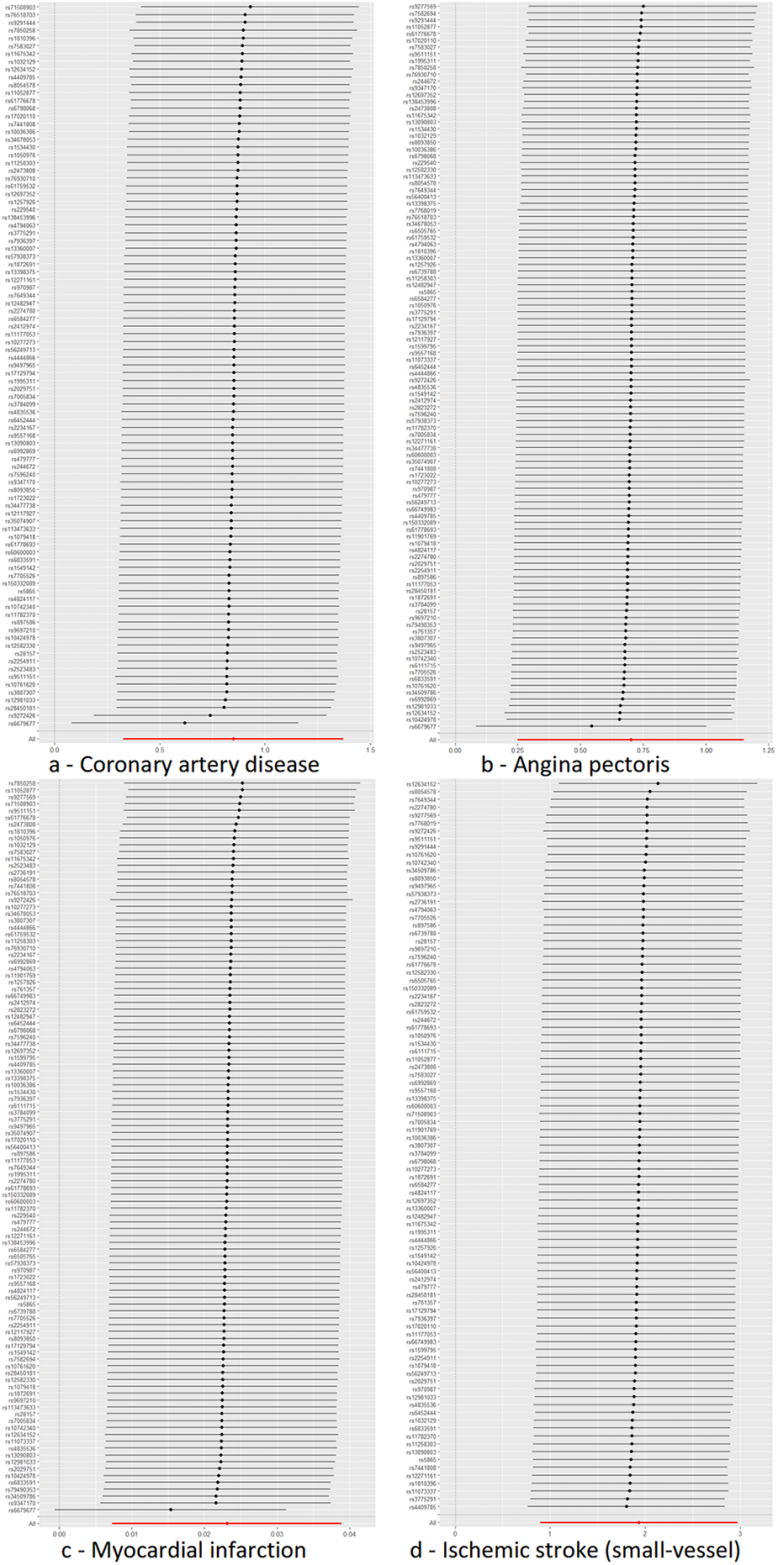
MR leave-one-out sensitivity analysis plot. The *y*-axis indicates the sequential exclusion of individual SNPs, the *x*-axis represents the IVW-derived effect estimates. Each horizontal line's points show the beta coefficients, with the lines demarcating their confidence intervals. The red line reflects the aggregate estimate, illustrating that no single SNP disproportionately influences the MR findings, as all betas are to the right of zero. The exposure factors for part labels **(a–d)** are all hypothyroidism, with the outcome factors being: coronary artery disease (CAD), angina pectoris (AP), myocardial infarction (MI), and IS-small vessels (IS-SV).

Cochran's *Q* test to quantify heterogeneity using both MR Egger and IVW method. Horizontal Multiplicity of Test Levels Using MR-Egger Regression with Intercepts (Table 3), revealing no evidence of heterogeneity or pleiotropy (*P* > 0.05). We represented heterogeneity results for each MR analysis as funnel plots (Figure 5) and positive MR analysis results for CAD, AP, MI, and IS-SV as scatterplots (Figure 6). After repeating the MR-PRESSO global test, no outliers were detected. The “leave-one-out” sensitivity analysis confirmed that no single SNP disproportionately influenced the MR results, as visualized for CAD, AP, MI, and IS-SV (Figure 7). Collectively, these analyses support the robustness of our MR findings.

### 3.4 Reverse MR analysis

**Figure 8:**
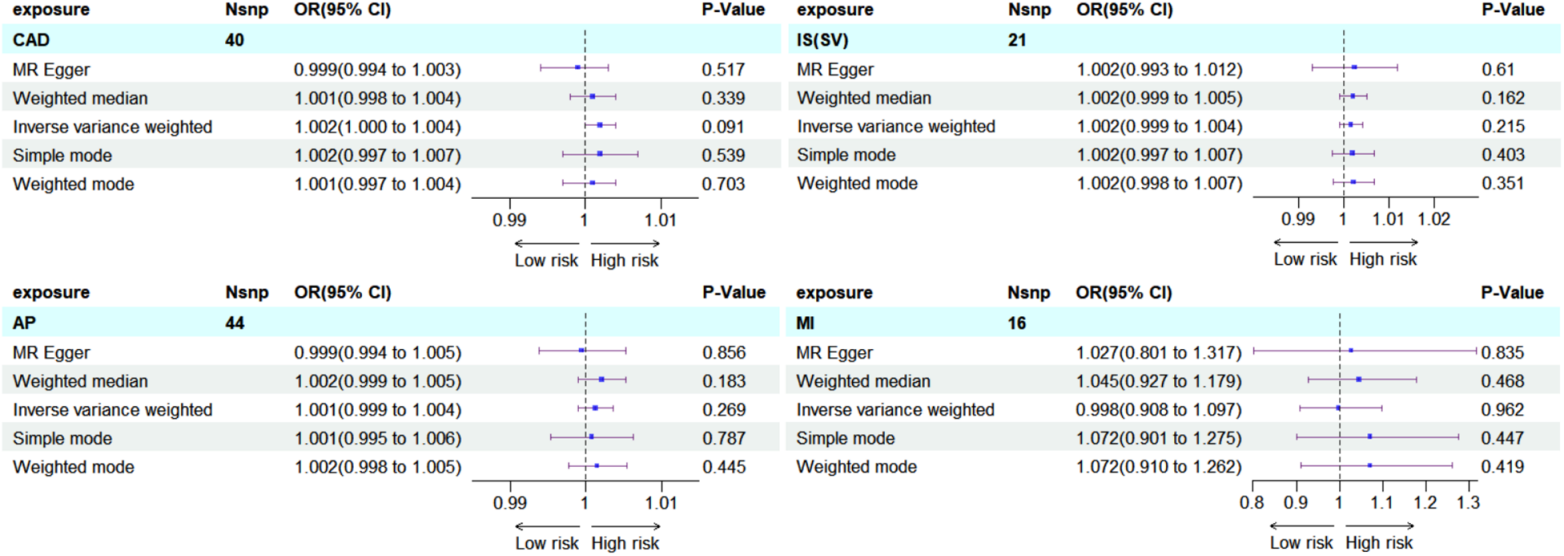
Subgroup forest plot for four exposure factors in reverse MR analysis, with rows shaded sky blue. The findings were consistently negative, reinforcing the unidirectional causal association between hypothyroidism and ASCVD.

To explore potential reverse causality, bidirectional MR analyses were conducted. These analyses involved using CAD, AP, MI, and IS-SV as exposures and hypothyroidism as the outcome, without altering the data source. The findings did not indicate any significant effect of these cardiovascular conditions on the risk of developing hypothyroidism, as shown in subgroup forest plots: CAD (OR = 1.002, 95%CI = 1.000–1.004, *P* = 0.091), AP (OR = 1.001, 95%CI = 0.999–1.004, *P* = 0.269), MI (OR = 0.998, 95%CI = 0.908–1.097, *P* = 0.962), IS-SV (OR* =* 1.002, 95%CI = 0.999–1.004, *P* = 0.215) (Figure 8).

### 3.5 Enrichment analysis

**Figure 9:**
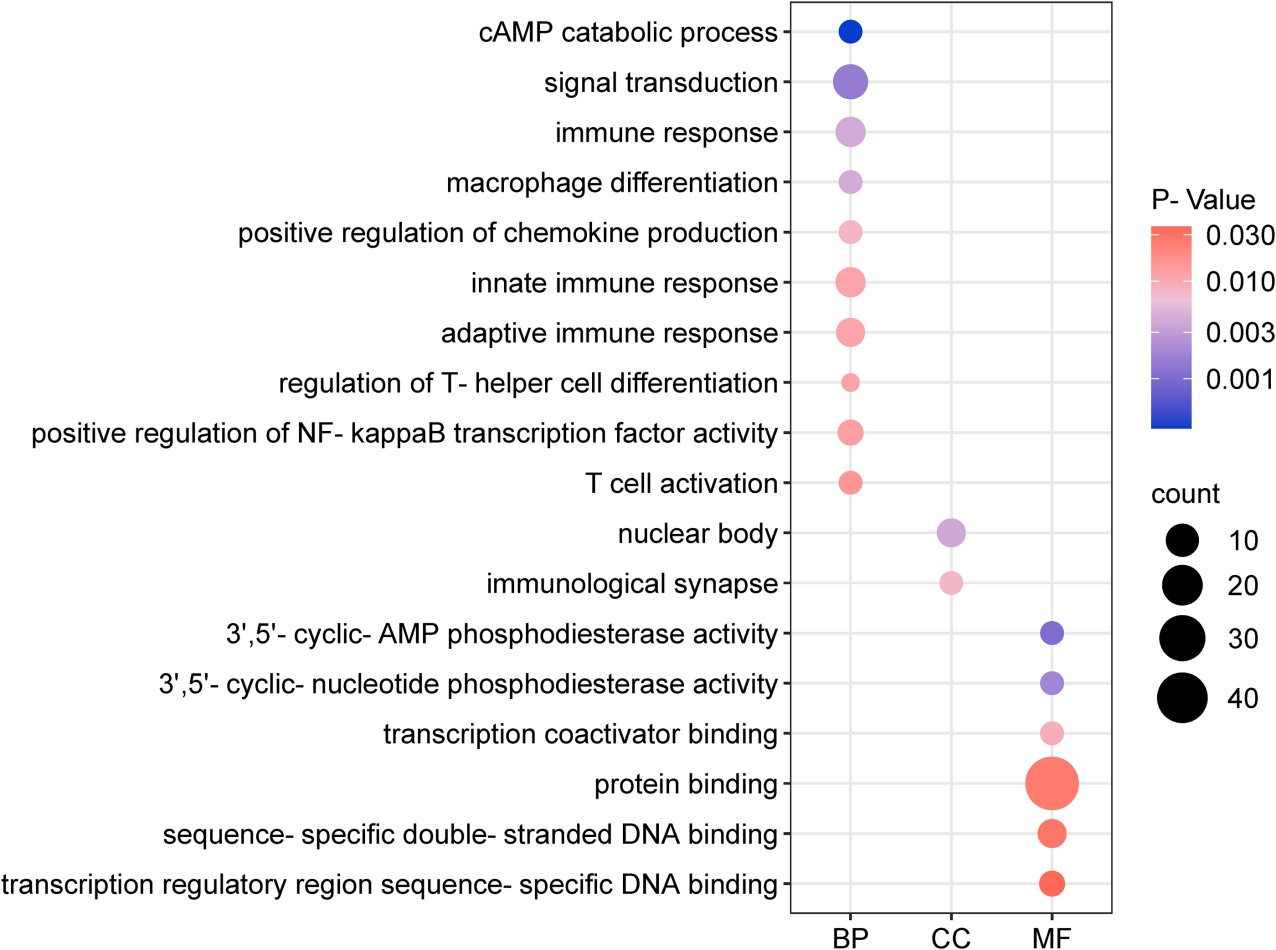
Go function enrichment analysis dot matrix plot. The *x*-axis categorizes BP, MF, and CC. Dot size depicts the count of enriched genes, and the color gradient represents the magnitude of enrichment *P*-values.

**Figure 10:**
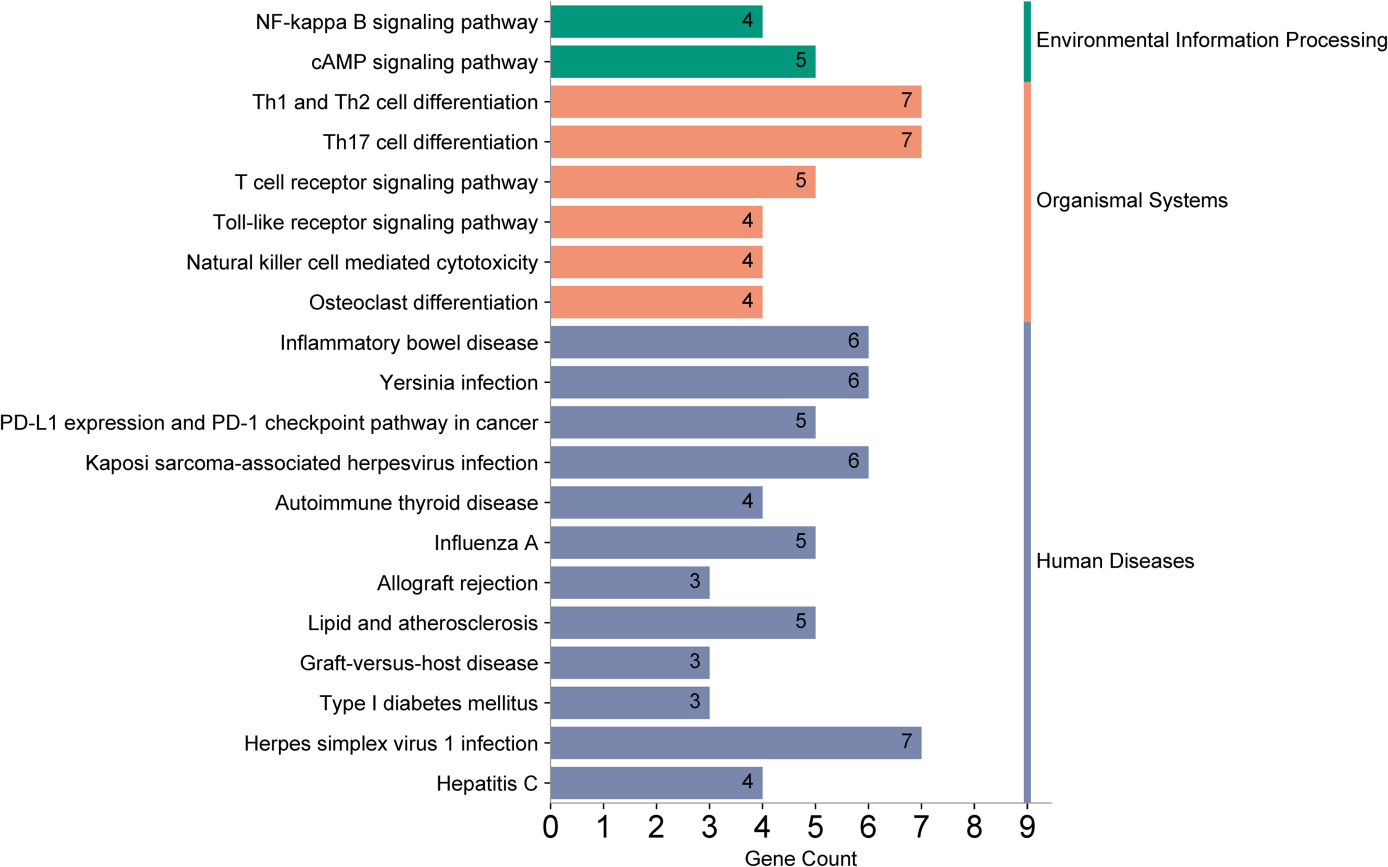
KEGG pathway enrichment analysis summary plot. The left *y*-axis lists the pathways enriched with genes, and the right *y*-axis categorizes them into secondary groups (environmental information processing, organismal systems, human diseases). The *x*-axis counts the number of genes enriched within each pathway.

Using the outcome of CAD (where MR analysis showed positive association), we located 205 neighboring genes around the SNPs through the “vautils” R package (setting: flanking = 50 kb, build=“hg19”, collapse = T). In instances of multiple neighboring genes per SNP, the closest one was selected. This resulted in a list of 83 neighboring genes, with an instance of overlap (rs2523483 and rs76518703 both corresponding to gene MIR6891) observed (Table 4). This gene list was analyzed using the DAVID database, selecting “Homo Sapiens” as the species and “official gene symbol” for gene identification. The analyses for GO enrichment in BP, MF, and CC, along with KEGG pathway enrichment, were conducted with default parameter settings. The enrichment analysis revealed that the cAMP catabolic process had the lowest *P*-value (*P* = 3.10 × 10^−4^), while protein binding had the largest number of enriched genes (count = 47). The GO functional enrichment analysis matrix dot plots (Figure 9) and the KEGG pathway enrichment secondary classification summary plot (Figure 10) displayed the significant findings (*P* < 0.05), highlighting “Th1 and Th2 cell differentiation” as the pathway with the lowest enrichment *P*-value (*P* = 2.20 × 10^−6^) and along with “Th17 cell differentiation” and “Herpes simplex virus 1 infection”, having the greatest gene enrichment count (count = 7).

**Table 4:** Neighboring genes of SNPs.

SNP	Chr	POS	GENE	SNP	Chr	POS	GENE
rs1995311	1	19820119	CAPZB	rs10277273	7	4785129	MIR4656
rs12117927	1	236629134	EDARADD	rs3807307	7	128579202	TNPO3
rs17129794	1	67794918	IL12RB2	rs897586	8	128193294	LINC01245
rs2029751	1	200698286	LOC101929224	rs6992869	8	61395832	RAB2A
rs61778693	1	38651781	LOC339442	rs11782370	8	23370018	SLC25A37
rs1723022	1	167405418	POU2F1	rs1810396	8	133918769	TG
rs2473808	1	19638883	PQLC2	rs1032129	8	119951900	TNFRSF11B
rs6679677	1	114303808	RSBN1	rs7005834	8	134214204	WISP1
rs61776678	1	38377021	SF3A3	rs970987	9	21585265	MIR31HG
rs2234167	1	2494330	TNFRSF14	rs34477738	9	5447227	PLGRKT
rs17020110	1	108354156	VAV3	rs2274780	9	127075021	PSMB7
rs13398375	2	8451701	LINC00299	rs9697210	9	131468740	ZER1
rs1534430	2	12644736	LOC100506457	rs11258303	10	6405534	LOC399715
rs7596240	2	242444173	STK25	rs71508903	10	63779871	MIR548AV
rs11675342	2	1407628	TPO	rs6584277	10	101278055	NKX2-3
rs5865	2	98373006	ZAP70	rs10761620	10	64057202	RTKN2
rs150332089	3	5011335	BHLHE40	rs7936397	11	577534	SCT
rs2254911	3	121828837	CD86	rs12271161	11	116979911	SIK3
rs57938373	3	39336038	CX3CR1	rs10742340	11	35317712	SLC1A2
rs12634152	3	188121019	LPP	rs479777	11	64107477	TRMT112
rs6798068	3	108162362	MYH15	rs11052877	12	9905690	CLECL1
rs7649344	3	37006396	TRANK1	rs11177053	12	68499237	IFNG
rs4444866	4	40307533	CHRNA9	rs76930710	12	68434459	IFNG-AS1
rs9291444	4	10713674	CLNK	rs12582330	12	103892941	LOC101929084
rs6833591	4	123546282	IL21-AS1	rs9511151	13	24786576	SPATA13-AS1
rs113473633	4	103449131	NFKB1	rs9557168	13	99807350	UBAC2-AS1
rs28450181	4	87819369	SLC10A6	rs34678053	14	106135271	MIR8071-2
rs3775291	4	187004074	TLR3	rs3784099	14	68749927	RAD51B
rs28157	5	102595837	C5orf30	rs1872691	16	50350210	MIR6771
rs13360007	5	156577720	MED7	rs138453996	16	67349478	SLC9A5
rs10036386	5	76543603	PDE8B	rs4794063	17	45804494	TBX21
rs6452444	5	71685736	PTCD2	rs61759532	17	7240391	YBX2
rs12697352	5	35837234	SPEF2	rs56249713	18	67533332	DOK6
rs244672	5	133419283	TCF7	rs8093850	18	77178302	NFATC1
rs7705526	5	1285974	TERT	rs1549142	19	18383794	PDE4C
rs9272426	6	32605189	HLA-DRB1	rs35074907	19	10600418	S1PR5
rs1050976	6	408079	IRF4	rs10424978	19	4837557	TICAM1
rs2523483	6	31353792	MIR6891	rs12981033	19	50197406	TSKS
rs76518703	6	31329466	MIR6891	rs12482947	21	43852037	UBASH3A
rs1079418	6	166047034	PDE10A	rs2412974	22	30539821	HORMAD2
rs9347170	6	167405187	RNASET2	rs4824117	22	50895133	SBF1
rs60600003	7	37382465	ELMO1	rs229540	22	37591290	SSTR3

## 4 Discussion

### 4.1 MR analysis

In this study, we employed Two-Sample MR using publicly available GWAS summary data to scrutinize the causal link between hypothyroidism and ASCVD. The findings suggest a genetic underpinning for the causal relationship between hypothyroidism and an increased risk of CAD, AP, MI, and IS-SV, offering novel insights for clinical prevention and therapeutic approaches. Moreover, for those with positive results in the forward Mendelian randomization analysis, a reverse analysis was conducted, which did not find a causal effect of ASCVD on hypothyroidism.

MR is an epidemiological tool that utilizes genetic variations as proxies to estimate the influence of modifiable exposures on disease outcomes, thereby inferring causality (23). We rigorously selected 122 SNPs associated with hypothyroidism and there was no weak instrumental variable bias. The selection criteria for each GWAS were based on the largest sample size and the most recent studies. The primary analysis relied on the IVW method, leveraging the inverse of variance for fitting without an intercept, which minimizes bias (24). In this MR study, the genetic variants used as instrumental variables were derived from multiple GWAS. Given the diversity in outcome definitions across these studies, we acknowledge that this may introduce a degree of heterogeneity. To address this, Cochrane's Q test was utilized to evaluate heterogeneity in the MR findings. Causal association reliability was assessed using the MR-Egger intercept, MR-PRESSO tests, and leave-one-out analysis, suggesting a consistent causal estimate. These analyses confirmed that our findings remain consistent despite potential heterogeneity in outcome definitions.

### 4.2 Enrichment analysis

GO enrichment analysis indicated the “cAMP catabolic process” had the most significant *P*-value. cAMP is a critical second messenger involved in cardiovascular regulation, influencing gene expression, cell morphology, and function. Its role is pivotal in modulating the contraction/relaxation of vascular smooth muscle cellsand vascular endothelial permeability (25). Moreover, KEGG enrichment analysis revealed that the “Th1 and Th2 cell differentiation” pathway had the most significant enrichment *P*-value and the largest number of associated genes. These T cell subsets are crucial for maintaining immunological homeostasis; their imbalance is implicated in autoimmunity and inflammation. Heightened Th1 levels are associated with inflammatory infiltration, vascular endothelial proliferation, platelet aggregation, and accelerated atherosclerosis (26). The “Th17 cell differentiation” pathway was also notable for its gene richness. Th17 cells, through IL-17A secretion, promote platelet adhesion and contribute to AP and CAD progression. Modulating the Th17/Treg ratio may offer a novel intervention to prevent or treat CAD in hypothyroid patients (26).

### 4.3 Correlation analysis

Clinical evidence positions hypothyroidism as a significant risk factor for ASCVD, correlating with increased all-cause cardiovascular mortality (27). An analysis by Rodondi N et al., encompassing 50,953 euthyroid participants and 3,348 with subclinical hypothyroidism (SCH) across seven countries, found that SCH notably elevates CAD mortality (28), especially with TSH levels ≥10.0 mIU/L. Hypothyroidism prevalence peaks in adults over 45, who concurrently face heightened ASCVD risk. Upon adjusting for confounders such as age, sex, BMI, hypertension, diabetes, and lipid levels, TSH was positively associated with coronary artery lesion severity and involved branches. Clinical studies underscore that cardiovascular risk persists even after optimal management of known risk factors (12), aligning with our findings.

Clinical treatment options remain contentious. BMJ (29) guidelines advise against T4 replacement therapy for subclinical hypothyroidism, barring specific conditions, due to the negligible impact on cardiovascular events. Conversely, a JAMA (10) review suggests T4 therapy for elevated TSH in subclinical hypothyroidism may decrease coronary heart disease mortality. Another study indicate T4 therapy might reduce adverse cardiovascular events post-percutaneous coronary intervention (30). As human genetics and gene and metabolomics, proteomics and transcriptomics data complement each other, the exploration of the causal relationship of the disease seen in this TSMR study will help to discover new biomarkers for predicting the occurrence of ASCVD or cardiovascular events, and to screen for possible therapeutic targets, which will be able to bring new ideas and directions for answering the controversial questions of clinical diagnosis and treatment.

### 4.4 Strengths and limitations of the study

This investigation first applies MR to delineate a causal association between hypothyroidism and ASCVD, confirming hypothyroidism as a risk factor for CAD, AP, MI, and IS-SV. It contributes significantly to clinical prevention and the management of these conditions. The MR approach mitigates confounding factors, a common issue in observational studies, and stringent criteria for data selection, instrumental variable selection, and sensitivity analyses enhance the study's validity.

However, limitations persist. In our study, we observed a strong causal association between the genetic variants and IS-SV, while the associations with other stroke subtypes were close to zero. This finding may be attributed to potential biological differences between IS-SV and other stroke types, as IS-SV is typically associated with microvascular pathology. It may also be due to chance, arising from the limitations of this type of research. To address this, we conducted sensitivity analyses, including leave-one-out analysis and MR-Egger regression, which confirmed the robustness of the IS-SV association. Future studies should replicate these findings in larger datasets to further validate this potential causal link. Additionally, the MR framework cannot account for socio-environmental factors. The primary reliance on European-derived GWAS data may reduce population stratification bias but limits the applicability of findings across diverse populations. Despite these constraints, the study contributes valuable insights to the field.

## 5 Conclusion

In our investigation, we employed two-sample MR methods to examine the causal relationship between hypothyroidism and seven types of ASCVD, utilizing aggregated data from GWAS. Our analysis confirms a heightened risk of CAD, AP, MI, and IS-SV associated with hypothyroidism. Given these findings, we advocate for the systematic cardiovascular screening of hypothyroidism patients for CAD, AP, MI, and IS-SV. A comprehensive treatment strategy is imperative to mitigate progression, enhance patients’ quality of life, and improve survival outcomes.

## Data Availability

The original contributions presented in the study are included in the article/Supplementary Material, further inquiries can be directed to the corresponding author.

## References

[1] Byrne RA Rossello X Coughlan JJ Barbato E Berry C Chieffo A 2023 ESC guidelines for the management of acute coronary syndromes. G Ital Cardiol (Rome). (2024) 25(2 Suppl 2):e1–112. 10.1714/4191.41785 38291910

[2] Blaha MJ DeFilippis AP. Multi-ethnic study of atherosclerosis (MESA). J Am Coll Cardiol. (2021) 77(25):3195–216. 10.1016/j.jacc.2021.05.006 34167645 PMC8091185

[3] Feske SK. Ischemic stroke. Am J Med. (2021) 134(12):1457–64. 10.1016/j.amjmed.2021.07.027 34454905

[4] Montaño A Hanley DF Hemphill JC 3rd., Hemorrhagic stroke. Handb Clin Neurol. (2021) 176:229–48. 10.1016/B978-0-444-64034-5.00019-5 33272397

[5] Prados-Torres A Poblador-Plou B Gimeno-Miguel A Calderón-Larrañaga A Poncel-Falcó A Gimeno-Feliú LA Cohort profile: the epidemiology of chronic diseases and multimorbidity. The EpiChron cohort study. Int J Epidemiol. (2018) 47(2):382–4f. 10.1093/ije/dyx259 29346556 PMC5913592

[6] Jabbar A Pingitore A Pearce SH Zaman A Iervasi G Razvi S. Thyroid hormones and cardiovascular disease. Nat Rev Cardiol. (2017) 14(1):39–55. 10.1038/nrcardio.2016.174 27811932

[7] De Vito P Incerpi S Pedersen JZ Luly P Davis FB Davis PJ. Thyroid hormones as modulators of immune activities at the cellular level. Thyroid. (2011) 21(8):879–90. 10.1089/thy.2010.0429 21745103

[8] Razvi S Jabbar A Pingitore A Danzi S Biondi B Klein I Thyroid hormones and cardiovascular function and diseases. J Am Coll Cardiol. (2018) 71(16):1781–96. 10.1016/j.jacc.2018.02.045 29673469

[9] Forini F Nicolini G Kusmic C Iervasi G. Protective effects of euthyroidism restoration on mitochondria function and quality control in cardiac pathophysiology. Int J Mol Sci. (2019) 20(14):3377. 10.3390/ijms20143377 31295805 PMC6678270

[10] Biondi B Cappola AR Cooper DS. Subclinical hypothyroidism: a review. JAMA. (2019) 322(2):153–60. 10.1001/jama.2019.9052 31287527

[11] Paschou SA Bletsa E Stampouloglou PK Tsigkou V Valatsou A Stefanaki K Thyroid disorders and cardiovascular manifestations: an update. Endocrine. (2022) 75(3):672–83. 10.1007/s12020-022-02982-4 35032315

[12] Gencer B Rodondi N. Should we screen for hypothyroidism in patients with cardiovascular disease? Eur Heart J. (2016) 37(26):2066–8. 10.1093/eurheartj/ehv694 26763791

[13] Smith GD Hemani G. Mendelian randomization: genetic anchors for causal inference in epidemiological studies. Hum Mol Genet. (2014) 23(R1):R89–98. 10.1093/hmg/ddu328 25064373 PMC4170722

[14] Bowden J Del Greco MF Minelli C Zhao Q Lawlor DA Sheehan NA Improving the accuracy of two-sample summary-data Mendelian randomization: moving beyond the NOME assumption. Int J Epidemiol. (2019) 48(3):728–42. 10.1093/ije/dyy258 30561657 PMC6659376

[15] Burgess S Bowden J Fall T Ingelsson E Thompson SG. Sensitivity analyses for robust causal inference from Mendelian randomization analyses with multiple genetic variants. Epidemiology. (2017) 28(1):30–42. 10.1097/EDE.0000000000000559 27749700 PMC5133381

[16] Bowden J Davey Smith G Haycock PC Burgess S. Consistent estimation in Mendelian randomization with some invalid instruments using a weighted median estimator. Genet Epidemiol. (2016) 40(4):304–14. 10.1002/gepi.219655 27061298 PMC4849733

[17] Bowden J Davey Smith G Burgess S. Mendelian randomization with invalid instruments: effect estimation and bias detection through egger regression. Int J Epidemiol. (2015) 44(2):512–25. 10.1093/ije/dyv080 26050253 PMC4469799

[18] Zhu J Zhou D Wang J Yang Y Chen D He F Frailty and cardiometabolic diseases a bidirectional Mendelian randomization study. Age Ageing. (2022) 51(11):afac256. 10.1093/ageing/afac256 36346739

[19] Ong JS MacGregor S. Implementing MR-PRESSO and GCTA-GSMR for pleiotropy assessment in Mendelian randomization studies from a practitioner's perspective. Genet Epidemiol. (2019) 43(6):609–16. 10.1002/gepi.22207 31045282 PMC6767464

[20] Tang D Chen M Huang X Zhang G Zeng L Zhang G SRplot: a free online platform for data visualization and graphing. PLoS One. (2023) 18(11):e0294236. 10.1371/journal.pone.0294236 37943830 PMC10635526

[21] Harshfield EL Georgakis MK Malik R Dichgans M Markus HS. Modifiable lifestyle factors and risk of stroke. stroke. (2021) 52(3):931–6. 10.1161/STROKEAHA.120.031710 33535786 PMC7903981

[22] Marx N Federici M Schütt K Müller-Wieland D Ajjan RA Antunes MJ 2023 ESC guidelines for the management of cardiovascular disease in patients with diabetes. Eur Heart J. (2023) 44(39):4043–140. 10.1093/eurheartj/ehad192 37622663

[23] Long Y Tang L Zhou Y Zhao S Zhu H. Causal relationship between gut microbiota and cancers: a two-sample Mendelian randomisation study. BMC Med. (2023) 21(1):66. 10.1186/s12916-023-02761-6 36810112 PMC9945666

[24] Burgess S. Sample size and power calculations in Mendelian randomization with a single instrumental variable and a binary outcome. Int J Epidemiol. (2014) 43(3):922–9. 10.1093/ije/dyu005 24608958 PMC4052137

[25] Barlow CA Rose P Pulver-Kaste RA Lounsbury KM. Excitation-transcription coupling in smooth muscle. J Physiol. (2006) 570(Pt 1):59–64. 10.1113/jphysiol.2005.098426 16223758 PMC1464285

[26] Huo Y Feng Q Fan J Huang J Zhu Y Wu Y Serum brain-derived neurotrophic factor in coronary heart disease: correlation with the T helper (Th)1/Th2 ratio. T:h17./regulatory T (Treg) ratio, and major adverse cardiovascular events. J Clin Lab Anal. (2023) 37(1):e24803. 10.1002/jcla.24803 36510348 PMC9833972

[27] Ning Y Cheng YJ Liu LJ Sara JD Cao ZY Zheng WP What is the association of hypothyroidism with risks of cardiovascular events and mortality? A meta-analysis of 55 cohort studies involving 1,898,314 participants. BMC Med. (2017) 15(1):21. 10.1186/s12916-017-0777-9 28148249 PMC5289009

[28] Rodondi N den Elzen WP Bauer DC Cappola AR Razvi S Walsh JP Subclinical hypothyroidism and the risk of coronary heart disease and mortality. JAMA. (2010) 304(12):1365–74. 10.1001/jama.2010.1361 20858880 PMC3923470

[29] Bekkering GE Agoritsas T Lytvyn L Heen AF Feller M Moutzouri E Thyroid hormones treatment for subclinical hypothyroidism: a clinical practice guideline. Br Med J. (2019) 365:l2006. 10.1136/bmj.l2006 31088853

[30] Zhang M Sara JD Matsuzawa Y Gharib H Bell MR Gulati R Clinical outcomes of patients with hypothyroidism undergoing percutaneous coronary intervention. Eur Heart J. (2016) 37(26):2055–65. 10.1093/eurheartj/ehv737 26757789 PMC4940453

